# Engineering skyrmions in transition-metal multilayers for spintronics

**DOI:** 10.1038/ncomms11779

**Published:** 2016-06-03

**Authors:** B. Dupé, G. Bihlmayer, M. Böttcher, S. Blügel, S. Heinze

**Affiliations:** 1 Institute of Theoretical Physics and Astrophysics, University of Kiel, Leibnizstrasse 15, 24098 Kiel, Germany; 2 Peter Grünberg Institut (PGI-1) and Institute for Advanced Simulation (IAS-1), Forschungszentrum Jülich and JARA, 52425 Jülich, Germany

## Abstract

Magnetic skyrmions are localized, topologically protected spin structures that have been proposed for storing or processing information due to their intriguing dynamical and transport properties. Important in terms of applications is the recent discovery of interface stabilized skyrmions as evidenced in ultra-thin transition-metal films. However, so far only skyrmions at interfaces with a single atomic layer of a magnetic material were reported, which greatly limits their potential for application in devices. Here we predict the emergence of skyrmions in [4*d*/Fe_2_/5*d*]_*n*_ multilayers, that is, structures composed of Fe biatomic layers sandwiched between 4*d* and 5*d* transition-metal layers. In these composite structures, the exchange and the Dzyaloshinskii–Moriya interactions that control skyrmion formation can be tuned separately by the two interfaces. This allows engineering skyrmions as shown based on density functional theory and spin dynamics simulations.

## Abstract (web summary)

Materials possessing anisotropic spin exchange interactions can support skyrmion quasiparticle spin textures, which may be exploited in nanomagnetic devices. Here, the authors predict the appearance of skyrmions in multilayered thin films of biatomic Fe sandwiched between 4*d* and 5*d* transition metals.

## Graphical abstract

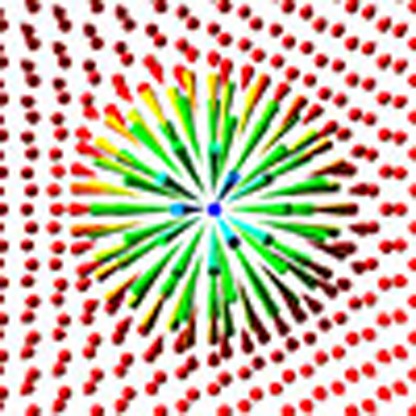

Topologically protected spin structures, in particular magnetic skyrmions^1^,^2^,^3^, have recently received much attention due to their promising applications in spintronics^4^,^5^,^6^. Among the different mechanisms that can induce a topologically non-trivial magnetic structure, the relativistic Dzyaloshinskii–Moriya interaction (DMI)^7^,^8^ arising in non-centrosymmetric systems is arguably the most prominent one^9^. Magnetic skyrmions have been first revealed in a few non-centrosymmetric bulk magnets, such as the chiral MnSi^10^,^11^ or FeCoSi^12^. In two-dimensional systems, the DMI arises due to the broken inversion symmetry at the interface between a magnetic film and a substrate^13^,^14^,^15^. For example, an Fe monolayer on an Ir(111) substrate exhibits strong DMI and on this system a nanoscale skyrmion lattice was first observed by scanning tunnelling microscopy^16^. This skyrmion lattice is stabilized by the short-range four-spin interaction and slight modifications of the film composition, for example, adding an atomic Pd overlayer, can push this system into a regime where individual skyrmions can exist, which can be manipulated by spin-polarized currents in scanning tunnelling microscopy^17^.

The manipulation of ultra-thin films such as Fe/Ir(111) is a versatile route to tailor properties such as the size or magnetic field needed to induce a magnetic skyrmion^18^,^19^. For practical applications, however, there are several difficulties that have to be overcome: Manipulation of skyrmions by electric currents^6^,^20^,^21^,^22^,^23^,^24^ and measurement of the topological Hall effect^25^,^26^,^27^ requires that a substantial fraction of the current runs through the magnetic structure. In a single Fe layer on a metallic substrate, this is difficult to realize. Creating large-area single Fe layers on a substrate requires specialized preparation methods that hamper large-scale production of these structures. Temperature stability and optimization of the size of these magnetic structures requires a tuning of different parameters such as exchange interactions, the DMI and the magnetic anisotropy.

Here we propose multilayers composed of Fe bilayers epitaxially sandwiched between 4*d* and 5*d* transition-metal (TM) layers as promising systems towards solving these issues. The repetition of layered structures is a powerful method in spintronics to engineer materials properties^28^,^29^,^30^: besides increasing the magnetic material in [4*d*/Fe/5*d*]_*n*_ multilayers, such structures have the key advantage that the essential magnetic interactions, the exchange and the DMI, can be controlled by two separate interfaces. Thereby, the requirement of pseudomorphic growth is limited to the interface, which induces the DMI, whereas intermixing at the other interface is not crucial. Based on density functional theory (DFT), we show a route to engineer the skyrmion systems by varying the interface composition. We focus on an Fe bilayer sandwiched between Ir and Rh/Pd layers; however, other combinations of 4*d* and 5*d* TMs are also feasible. By changing the 4*d* band filling—here by alloying Rh with Pd—the exchange interaction in the adjacent Fe layers can be tuned due to strong hybridization at the interface^31^. The 5*d* TM Ir, on the other hand, ensures a strong DMI. Using spin dynamics simulations, we find the emergence of skyrmions in the Fe bilayers and study their properties.

## Results

### First-principles calculations

To access the magnetic interactions in TM multilayers, we have performed DFT calculations for various non-collinear spin structures including spin–orbit coupling^32^,^33^. By mapping the total energies of the different magnetic configurations to an extended Heisenberg model, we can extract parameters for the interactions. We consider homogeneous flat spin spirals with a period *λ*, in which the magnetization rotates along a given crystallographic direction that lies in the plane of the interface by an angle of *ϕ*=(2*π*/*λ*)*a* from atom to atom, where *a* is the spacing between adjacent atoms (see Methods and Supplementary Note 1). This approach has been successfully used in the past to study complex spin textures in a wide range of systems such as biatomic chains at surfaces^34^, ultra-thin film skyrmion systems^16^,^18^ and magnetic mono-15,35,36 and bilayers^37^.

A characteristic quantity of the exchange interaction in the considered TM multilayers is the effective nearest-neighbour exchange constant *J*_eff_. It is a measure of how quickly the energy rises for long-wave length spin spirals with respect to the ferromagnetic (FM) state. *J*_eff_ was obtained by fitting the dispersion curve *E*(**q**) of spin spirals characterized by the wave vector **q** in the vicinity of the FM state (|**q**|<0.1 × 2*π*/*a*) with a parabola 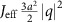 where *a* is the nearest-neighbour distance within the layers (for energy dispersions, see Supplementary Fig. 1). Therefore, a negative value of *J*_eff_ indicates a spin spiral ground state driven by exchange interaction. As a second characteristic quantity, we include the energy difference between a spin spiral state with a wavelength *λ*=2*π*/|*q*|=2.7 nm and the FM state, denoted by *E*_SS_.

**Figure 1:**
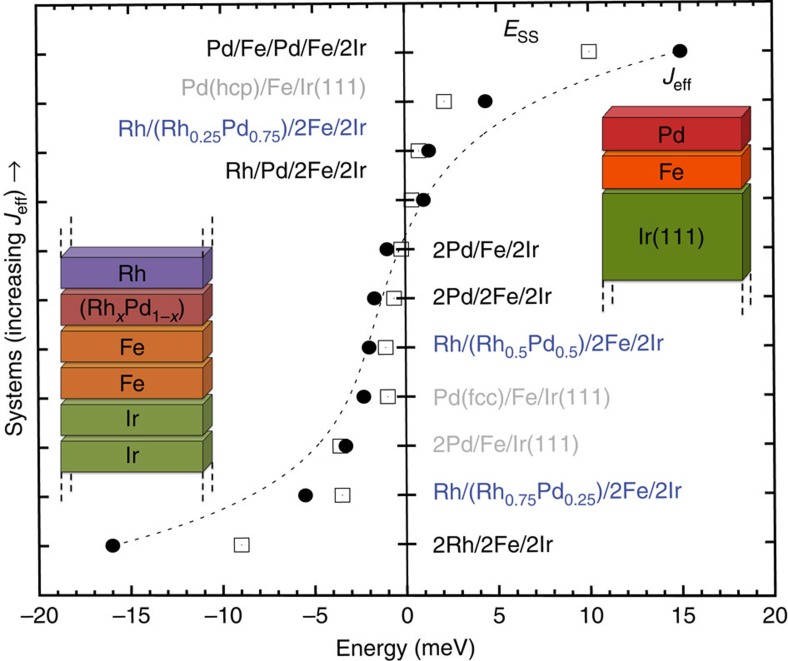
Effective exchange interaction in multilayers. *J*_eff_ (full circles) is given for TM multilayers (black/blue fonts) and ultra-thin films on surfaces (grey fonts). Insets show sketches of the multilayer and thin film geometry. The energy difference between a spin spiral with a wavelength of 2.7 nm with respect to the FM state, *E*_SS_, is given by empty squares. The dashed line is a guide for the eye.

*J*_eff_ and *E*_SS_ are displayed for various systems in Fig. 1. We start with the monolayer films on substrates, which can host single skyrmions as shown in experiment^17^ and explained by theory^18^. For both fcc and hcp stacking of the Pd overlayer on Fe/Ir(111), we find a rather small value of the effective exchange constant, that is, *J*_eff_=−2.3 and +4.4 meV, respectively. The DMI, which is of similar strength in both systems^18^, can stabilize skyrmions in these films in an applied magnetic field. It is noteworthy that from recent experiments on the field-dependent diameter of skyrmions in Pd/Fe/Ir(111), Romming *et al*.^38^ determined an exchange stiffness that corresponds approximately to a *J*_eff_ of 1.6 meV, which is very close to the values we find here. For two layers of Pd on Fe/Ir(111), *J*_eff_ is negative and slightly stronger than for Pd(fcc)/Fe/Ir(111) and a skyrmion phase also occurs in a magnetic field as found in our simulations. In these two systems, the spin spiral ground state is already enforced by the exchange interaction and becomes even more favourable due to the DMI. We can also conclude the spin spiral ground state from the negative value of *E*_SS_, which closely follows the trend of *J*_eff_.

Now we turn to the multilayers that are built by an epitaxial fcc stacking of (111) layers with a geometry as in an Ir bulk crystal but allowing for structural relaxation in the direction normal to the layers (see Methods and Supplementary Table 1). We first consider a multilayer, which is a repetition of a sandwich consisting of one atomic Fe layer between two Pd and two Ir layers denoted as 2Pd/Fe/2Ir. As shown in Fig. 1, the effective exchange interaction is very similar to that of the ultra-thin film system Pd(fcc)/Fe/Ir(111), which demonstrates that *J*_eff_ is mainly controlled by the interfaces. A first naive solution to increase the amount of magnetic material, that is, Fe layers in the sandwich, would be to repeat the Pd/Fe interface sequence as in Pd/Fe/Pd/Fe/2Ir. However, *J*_eff_ becomes much larger (≈15 meV), indicating strong FM exchange interaction, which prevents skyrmion formation. If, on the other hand, we consider the multilayer with a basic unit consisting of two layers of Pd, Fe and Ir, that is, 2Pd/2Fe/2Ir, the effective exchange interaction is of the same order as in the original single Fe-layer system.

We can understand the role of Pd by replacing it with Rh, which has one electron less in the 4*d* shell. For a multilayer consisting of 2Rh/2Fe/2Ir, we obtain a value of *J*_eff_≈−16 meV, that is, negative and of large absolute value. In this system, we find an exchange-driven spin spiral ground state and no skyrmion formation in a reasonable external magnetic field. By choosing an intermediate interface composition, for example, Rh/Rh_0.75_Pd_0.25_/2Fe/2Ir, we can increase the 4*d* band filling quasi continuously and thereby increase the value of the effective exchange coupling *J*_eff_. For a large range of interface alloy compositions Rh_*x*_Pd_1−*x*_, we find multilayers with a small *J*_eff_, which can host skyrmionic ground states as we show below using spin dynamics simulations.

### Spin dynamics simulations

After having revealed the feasibility to tune the magnetic interactions in TM multilayers, we demonstrate the formation of skyrmions in such systems. To explore the magnetic phase space in an external magnetic field, we employ the spin Hamiltonian


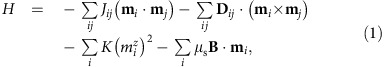


which describes the magnetic interactions between the magnetic moments **M**_*i*_ of atoms at sites **R**_*i*_ where **m**_*i*_=**M**_*i*_/*M*_*i*_. The parameters for the exchange interaction (*J*_*ij*_), the DMI (**D**_*ij*_), the magnetic moments (*μ*_s_), as well as an uniaxial magnetocrystalline anisotropy (*K*) were obtained from DFT.

In a magnetic bilayer, it is convenient to split the exchange interaction into an intralayer contribution, 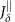, which corresponds to the exchange coupling to an atom of the *δ*-th nearest neighbour shell within an Fe layer, and the interlayer part 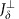, which corresponds to the coupling between the two Fe layers (see Supplementary Note 2 and Supplementary Table 2 for details). For the multilayer Rh/Pd/2Fe/2Ir, on which we focus below as a generic example, the interlayer coupling between nearest neighbours is very strong, 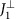
=24.73 meV, and favours FM alignment of magnetic moments of the two Fe layers. However, the nearest-neighbour exchange coupling within each Fe layer, 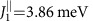, is much smaller and on the order of antiferromagnetic (AFM) exchange with third nearest neighbours, 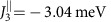. In total, the competition between intra- and interlayer exchange leads to the small effective exchange constant *J*_eff_=1 meV (see Fig. 1), which indicates a very flat dispersion curve close to the FM state (see Supplementary Fig. 2 and Supplementary Note 3). It is noteworthy that taking into account separate intralayer exchange coupling constants within the two Fe layers does not lead to any qualitative changes (see Supplementary Note 4, Supplementary Figs 3–6 and Supplementary Table 3).

The DMI is of primary importance to stabilize skyrmions and has to be carefully evaluated for the bilayer case. Although there are contributions from the two interfaces, we find from our DFT calculations that the DMI is controlled by the Fe interface to the heavy 5*d* material (see Supplementary Note 3 and Supplementary Fig. 2). Within our spin Hamiltonian, we can describe it by an effective nearest-neighbour **D**-vector acting on the Fe moments at the Fe/Ir interface only. We obtain a value of *D*=1.3 meV for Rh/Pd/2Fe/2Ir with very little variation on changing the composition of the Rh_*x*_Pd_1−*x*_ interface (on the order of 0.1 meV). This value is very similar to those obtained for the ultra-thin film system Pd/Fe/Ir(111), that is, *D*=1.0 and 1.35 meV for fcc and hcp stacking of Pd, respectively^18^. The strength of the DMI is sufficient to create a spin spiral ground state for Rh/Pd/2Fe/2Ir with a right-hand rotation sense and a period of *λ*=2.25 nm (*cf*. Supplementary Note 3). The obtained uniaxial anisotropy, *K*, is 0.6 meV per Fe atom and favours an out-of-plane magnetization.

**Figure 2:**
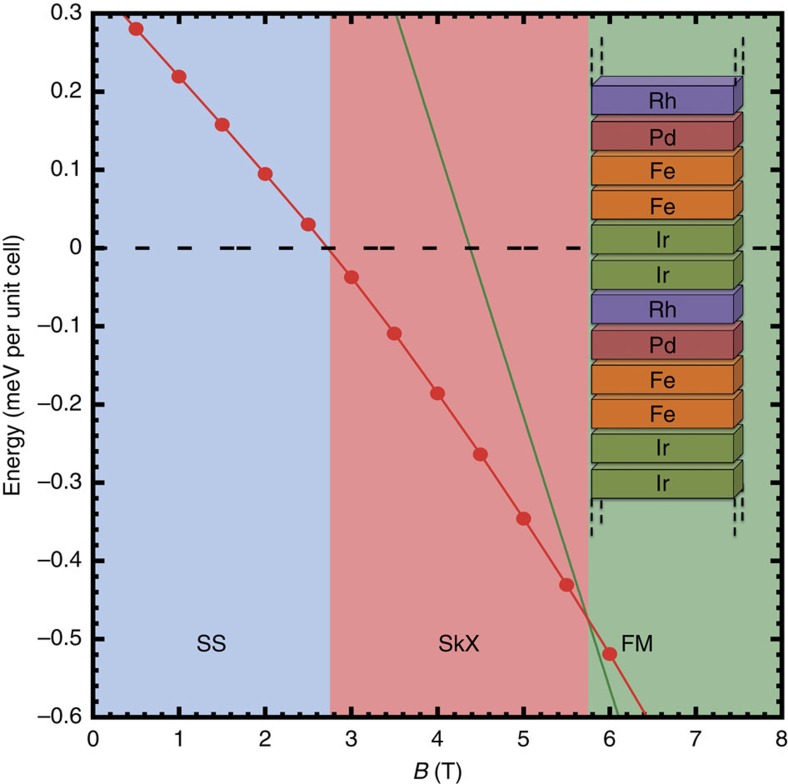
Low-temperature magnetic phase diagram of the multilayer Rh/Pd/2Fe/2Ir. The total energy of the FM state (green line) and of the skyrmion lattice (SkX, red line) is shown with respect to the spin spiral (SS) state (dashed black line). The regimes of the SS, SkX and FM phase are indicated by blue, red and green colour, respectively. The multilayer structure is shown in the inset.

We show the formation of skyrmions in multilayers by studying our spin Hamiltonian parametrized from first-principles using spin dynamics simulations (see Methods). In Fig. 2, the obtained low-temperature phase diagram is shown as a function of an external magnetic field applied perpendicular to the layers. At *B*=0 T, the lowest energy is obtained for a spin spiral with a period of *λ*=2.25 nm. As the magnetic field increases the spin spiral is destabilized and at *B*=2.75 T a transition to a skyrmion lattice occurs. Fields of this order of magnitude have been used, for example, in ref. 17, to induce skyrmions in monolayers experimentally. Further fine-tuning of the materials in the multilayer can be used to get to even lower critical fields. At *B*=5.8 T, the magnetic field becomes large enough to stabilize the FM state.

As the DMI does not vary much on changing the composition *x* of the Rh/Rh_*x*_Pd_1−*x*_/2Fe/2Ir multilayer and the influence of the magnetocrystalline anisotropy energy is rather moderate, the magnetic field strengths at the phase transitions depend mainly on the effective exchange interaction *J*_eff_. Although this interaction is influenced by factors such as alloying at the interfaces, it can be tuned as shown in Fig. 1, allowing a control over the phase diagram. (Limiting scenarios with respect to variation of the exchange interaction and magnetocrystalline anisotropy are discussed in the Supplementary Note 5 and shown in Supplementary Figs 7 and 8.)

**Figure 3:**
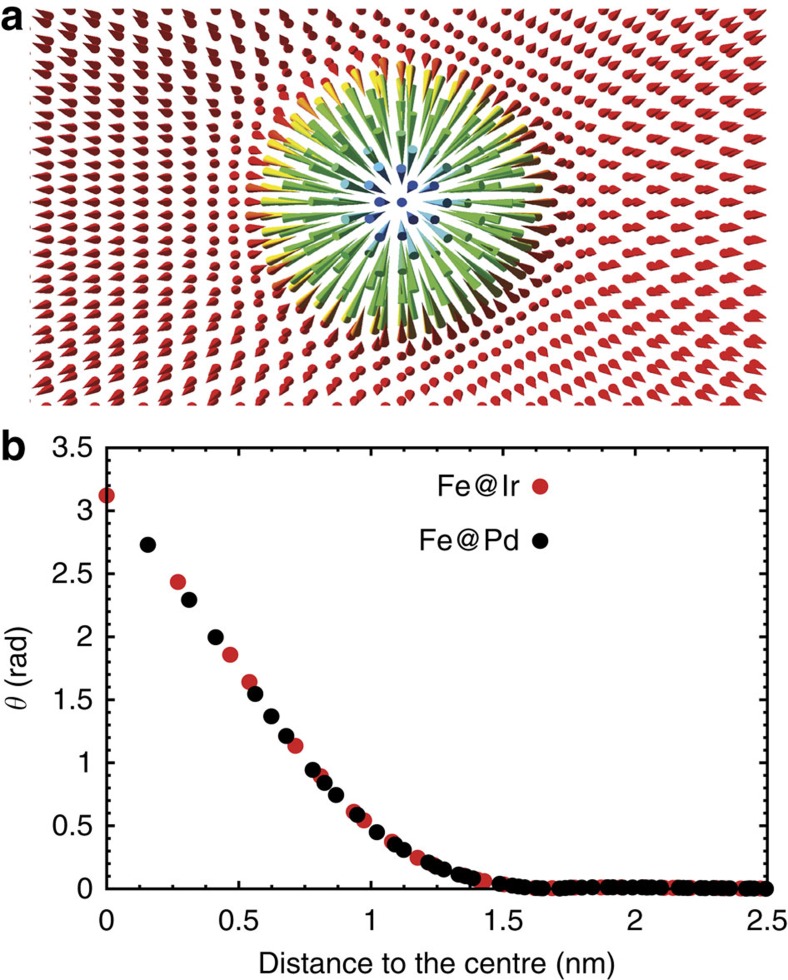
Skyrmion profile in the multilayer Rh/Pd/2Fe/2Ir. (**a**) Top view of the magnetization of the skyrmion with red arrows pointing up and green/blue arrows pointing down. (**b**) Magnetization profile of an isolated skyrmion at a magnetic field of *B*=3.5 T averaged at a given radial distance *r* from the centre. The polar angle *θ* of the magnetization vector is shown as a function of *r*. Red dots correspond to the Fe atoms at the Fe/Ir interface and the black dots correspond to the Fe atoms on the Pd side.

In Fig. 3, we present the profile of an isolated skyrmion in the bilayer at *B*=3.5 T, that is, the polar angle *θ* of the local magnetization as a function of the distance from the centre. The profile was obtained by imposing the theoretical profile^39^ and relaxing this spin structure within our spin dynamics simulation. The curves for both Fe layers show that at the centre of the skyrmion the magnetic moments are antiparallel to the surrounding FM background. We have obtained a skyrmion diameter defined as in ref. 39 of ∼2.2 nm, consistent with the spin spiral minimum.

**Figure 4:**
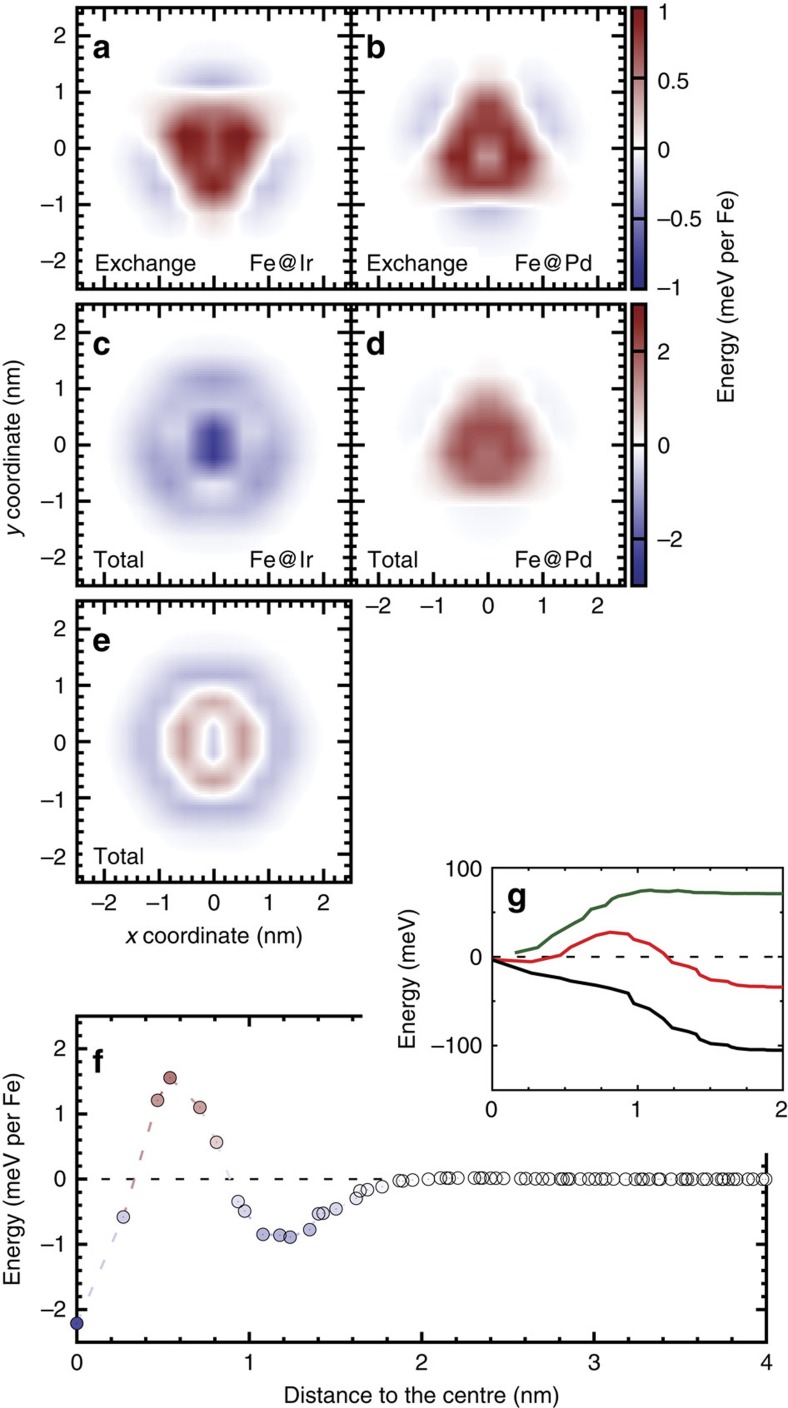
Site-resolved energy contributions for a skyrmion in the multilayer Rh/Pd/2Fe/2Ir. The isolated skyrmion has been obtained at a magnetic field of *B*=3.5 T (*cf*. skyrmion profile in Fig. 3). (**a**,**b**) The exchange energy in the Fe layer at the Ir interface (Fe@Ir) and at the Pd interface (Fe@Pd), respectively. (**c**,**d**) The total energy (exchange, DMI, Zeeman and anisotropy) for the two Fe layers and (**e**) for the complete Fe bilayer. The colour scale bar of the energy is given to the right of the panels, indicating stabilizing (*E*<0) and destabilizing (*E*>0) contributions. For (**e**), the scale bar is the same as for **c**,**d**. (**f**) Total energy per Fe atom averaged over the *δ*^th^ neighbour shell for **e**. The inset (**g**) shows the total energy summed over the shells from the skyrmion centre up to the radius *r* of the Fe bilayer (red), Fe@Pd (green) and Fe@Ir (black).

To understand the stabilization mechanism of a skyrmion in the Fe bilayer, we study the contributions of the different interactions to the site-resolved energy with respect to the FM background in each Fe layer for the isolated skyrmion at *B*=3.5 T. The site-resolved energy due to exchange (Fig. 4a,b) is very similar for the two Fe layers apart from a rotation by 60° due to the different position of the first nearest-neighbour atoms in the adjacent layer, which are coupled by 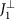 giving the main contribution to the exchange. The site-resolved exchange energy is minimal in the skyrmion centre where a moderate rotation of the magnetization (*cf*. Fig. 3) corresponding to a long wavelength spin spiral is energetically favoured. As we move away from the centre of the skyrmion, the canting between nearest-neighbour spins and the exchange energy increases, reaches a maximum at a distance of 1 nm from the core and decreases again when reaching the FM background.

The site-resolved total energy reveals the difference between the two Fe layers. For the Fe layer at the Pd interface (Fig. 4d) the DM interaction vanishes and the total energy is only increased by the Zeeman term and the anisotropy contribution. However, the Fe layer at the Ir interface (Fig. 4c) is subject to a significant DMI and its energy contribution outweighs the anisotropy and the Zeeman term, leading to a stabilizing (negative) energy within the entire skyrmion. The total site-resolved energy of the Fe bilayer, that is, the sum of the two layers, shown in Fig. 4e,f, exhibits a small local minimum at the centre of the skyrmion due to the compensation of the DMI from the Fe/Ir interface and the Zeeman and anisotropy terms. As we move away from the centre, the total energy first increases due to the strong exchange and Zeeman contributions and the rise of the anisotropy term, and decreases beyond ∼0.5 nm due to the DM term (*cf*. Fig. 4f).

The site-resolved energy summed over all shells within the radius *r* around the skyrmion centre is shown for the Fe bilayer and the two Fe layers in Fig. 4g. For the Fe layer at the Pd interface (green curve), the energy is always positive as expected from the site-resolved energy (*cf*. Fig. 4d) and saturates at ∼*r*=1.25 nm. For the Fe layer at the Ir interface (black curve), the energy is negative, indicating a skyrmion stabilization due to the DMI. The competition of these two contributions leads to the profile for the Fe bilayer (red curve), which displays a positive maximum at small radius but converges to a negative value of about −32 meV, indicating that the skyrmion is energetically favourable due to the DM contribution at the rim of the skyrmion.

## Discussion

We have focused on the system composed of an Fe bilayer sandwiched between Rh/Pd and Ir layers. For Fe monolayers on 4*d* and 5*d* TM surfaces, we have demonstrated before that the exchange interaction can be tuned by the substrate band filling from FM to AFM^31^,^40^,^41^. Therefore, other TM multilayers, for example, Ru_*x*_Pd_1−*x*_/2Fe/Pt, allow a similar tuning of *J*_eff_ by varying the composition *x*. The strength of the DMI, on the other hand, will be determined by the interface with the 5*d*-TM, for example, Ir or Pt^42^,^43^.

We have not considered to increase the number of Fe layers, for example, to trilayers, as this would increase the effective exchange interaction and reduce the effect of the two interfaces on the magnetic properties. The increase of magnetic material is ensured in our setup by the repetitions of the sandwich layers and strong coupling between skyrmions in adjacent bilayers. This leads to enhanced transition temperatures as shown in Supplementary Note 6 and Supplementary Fig. 9. TM multilayers consisting of a repetition of a few atomic layers as proposed here have recently been realized experimentally to stabilize chiral domain walls^29^.

In conclusion, we have shown that magnetic skyrmions can emerge in TM multilayers consisting of Fe bilayers sandwiched between 4*d* and 5*d* TMs. Such systems provide a rich field for the formation of skyrmions and allow to go beyond the monolayer systems discussed so far^16^,^17^,^18^ to a thickness range where the unique transport properties of these systems can be studied and exploited in devices. Recent success in tailoring the DMI at the interface to the 5*d* material^28^ can be combined with our concept to tune the exchange interaction by the choice of the 4*d* component of the multilayers, thus opening a route to engineer skyrmion properties for spintronic applications.

## Methods

### First-principles calculations

We have explored the multilayer structures from first-principles based on the full-potential linearized augmented plane wave method as implemented in the FLEUR code (www.flapw.de). Within this approach we can calculate the total energy of non-collinear magnetic structures such as spin spirals^32^ including the DMI in first-order perturbation theory with respect to the spin–orbit coupling^33^. We have used a two-dimensional hexagonal p(1 × 1) unit cell within each layer and the in-plane lattice parameter of the Ir(111) surface as obtained from DFT in ref. 18. The distances between the different layers were relaxed using the mixed functional suggested in ref. 44, which is ideally suited for interfaces of 3*d* and 5*d* TMs. The magnetic properties were obtained within the local density approximation^45^. To study alloying within the RhPd layers, we have used the virtual crystal approximation^46^, which has been used successfully for various types of systems from solid solutions^47^ to magnetic ultra-thin films^40^. Further computational details can be found in Supplementary Note 1.

### Spin dynamics simulations

To calculate the different energy differences between the FM, SkX and SS phases, we have relaxed a spin lattice of the bilayer with (100 × 100) spins in each layer according to the Landau–Lifshitz equation of spin dynamics:


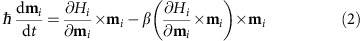


where *β* is the damping term and *H*_*i*_ is the Hamiltonian of spin *i* and is expressed by the Hamiltonian given in equation (1) if we omit the sum over *i*. To achieve fast relaxation, we have used *β*=0.95. We have carried out simulations on a nanosecond timescale with time steps ranging from 0.1 to 10 fs. The equation of motion was integrated with a simple Euler, a Heun and two semi-implicit integrators^48^ leading to the same results. To ensure that we reached the ground state, we have simultaneously run Monte Carlo simulations with a Metropolis algorithm on these configurations at *T*=0 K. Depending on the system, both techniques give similar results with a precision on the order of a few μeV per spin, sufficient to discriminate between the different phases in our cases.

### Data availability

The authors declare that the data supporting the findings of this study are available within the article and its Supplementary Information files.

## Additional information

**How to cite this article:** Dupé, B. *et al*. Engineering skyrmions in transition-metal multilayers for spintronics. *Nat. Commun.* 7:11779 doi: 10.1038/ncomms11779 (2016).

## Supplementary Material

Supplementary Information Supplementary Figures 1-9, Supplementary Tables 1-3, Supplementary Notes 1-6 and Supplementary References
