# Supplementary material for: Engineering skyrmions in transition-metal multilayers for spintronics
Source: Nat Commun. 2016 Jun 3;7:11779. doi: 10.1038/ncomms11779 (PMC4895799; doi:10.1038/ncomms11779)
Supplement: Supplementary Information — Supplementary Figures 1-9, Supplementary Tables 1-3, Supplementary Notes 1-6 and Supplementary References [file ncomms11779-s1.pdf]

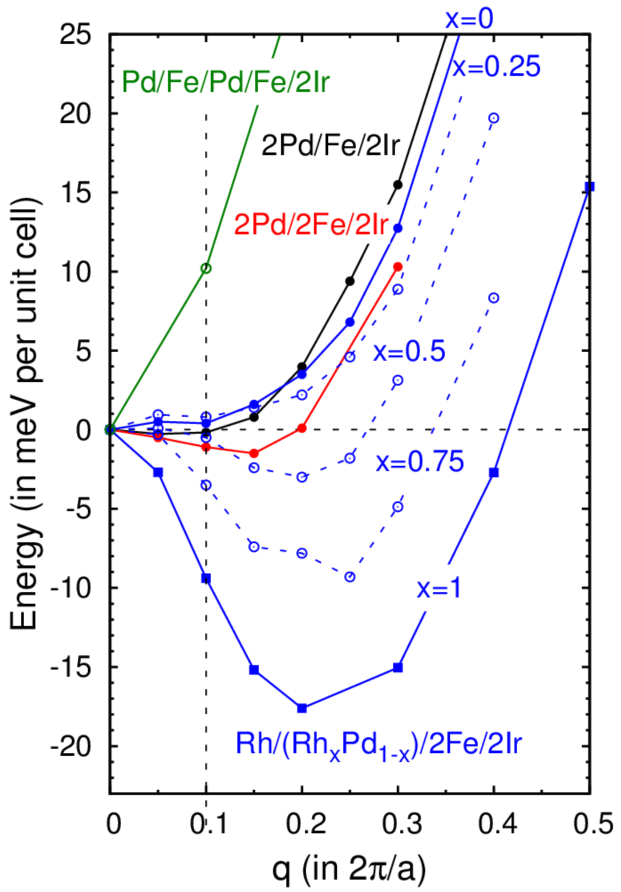

Supplementary Figure 1 | Energy dispersion $E(\mathbf{q})$ of spin spirals in the multilayer systems for $\mathbf{q}$ along the $\bar{\Gamma} - \bar{K}$ direction. The $\bar{K}$-point is located at $q = \frac{2}{3}$. The dashed line indicates the q-vector for which E_{SS} was calculated in Fig. 1 of the main text. Spin-orbit coupling has not been taken into account.

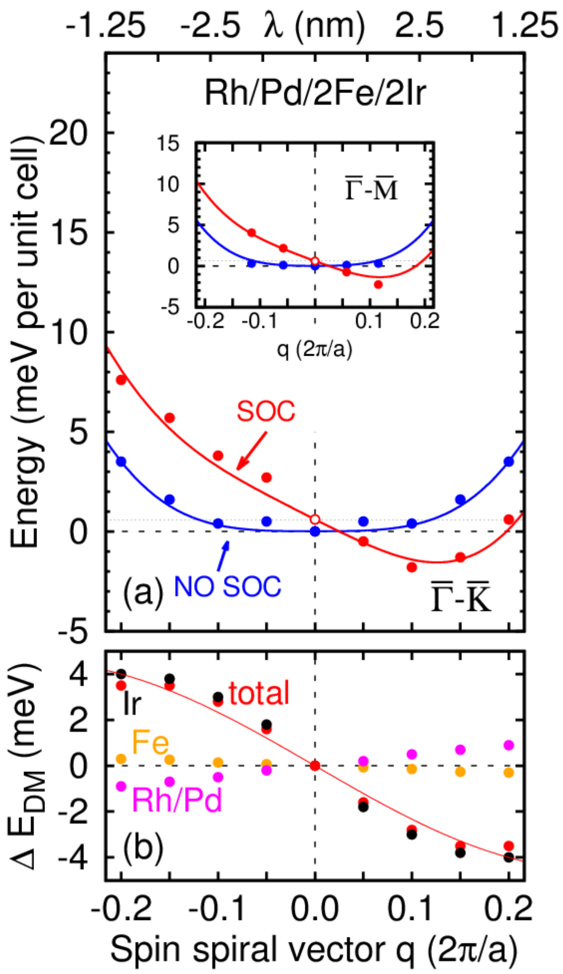

Supplementary Figure 2 | (a) Energy dispersion of spin spirals for the multilayer system Rh/Pd/2Fe/2Ir in the $\bar{\Gamma} - \bar{K}$ and in the $\bar{\Gamma} - \bar{M}$ (inset) directions without spin orbit coupling (SOC) (blue curves) and with SOC (red curves). (b) Decomposition of the SOC contribution to the energy originating from the Ir, Fe, and Rh/Pd layers and the total contribution.

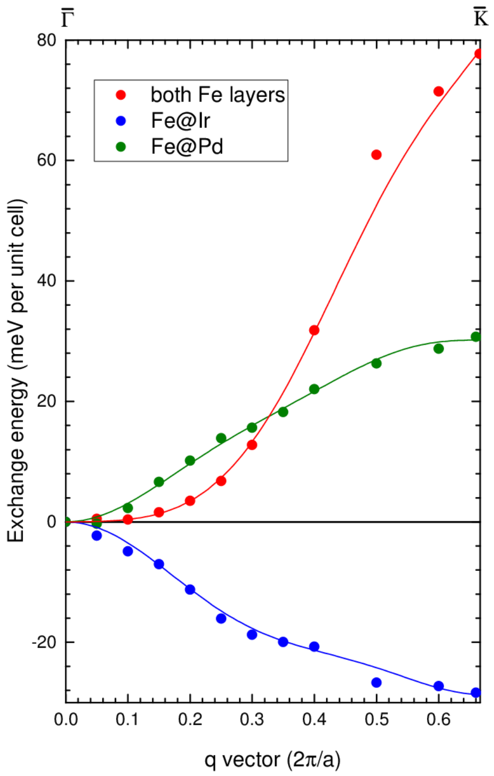

Supplementary Figure 3 | Energy dispersion curves of a homogeneous spin spiral propagating in the two Fe layers simultaneously (red), only in the Fe@Ir (blue) and only in the Fe@Pd (green). The data points were obtained by first principles calculations and the continuous lines were obtained by fitting the data points with an extended Heisenberg model as described in Supplementary Note 2. The coefficients are given in Supplementary Table 3.

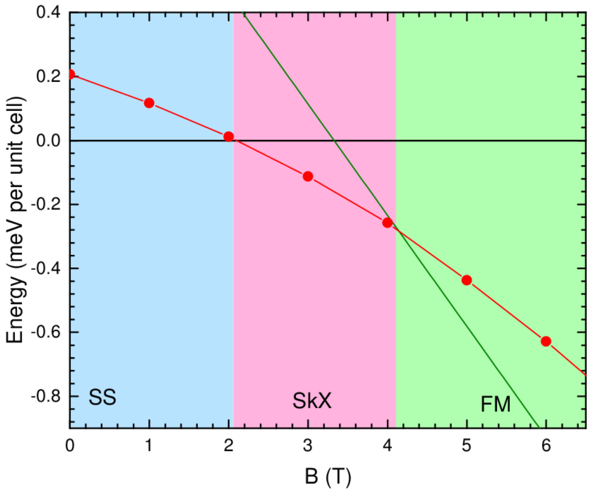

Supplementary Figure 4 | Low temperature magnetic phase diagram of the multilayer Rh/Pd/2Fe/2Ir assuming different intra-layer exchange couplings $J_{ij}^{\|}$ and DMI in the two Fe layers (see Supplementary Note 4 for details). The total energy of the ferromagnetic (FM) state (green line) and of the skyrmion lattice (SkX, red line) is shown with respect to the spin spiral (SS) state (dashed black line). The regimes of the SS, SkX, and FM phase are indicated by blue, red, and green color, respectively.

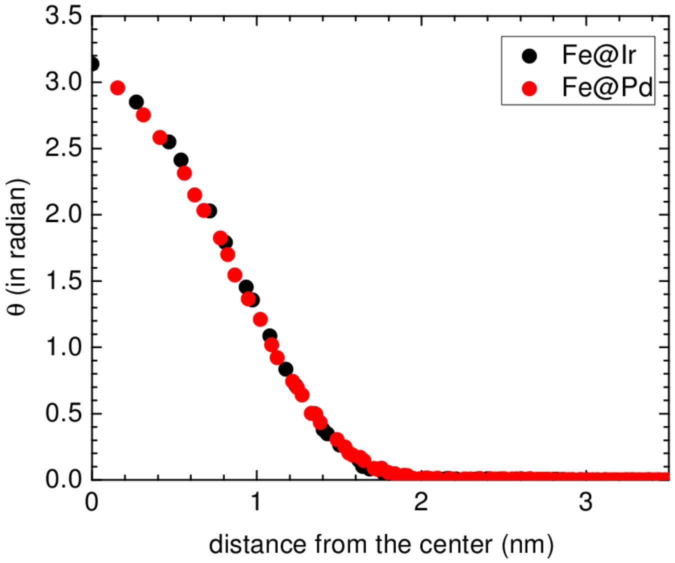

Supplementary Figure 5 | Magnetization profile of an isolated skyrmion in the multilayer Rh/Pd/2Fe/2Ir (assuming different intra-layer exchange couplings $J_{ij}^{\parallel}$ and DMI in the two Fe layers as discussed in Supplementary Note 4) averaged at a given radial distance r from the center. The polar angle θ of the magnetization vector is shown as a function of r. Red dots correspond to the Fe atoms at the Fe/Ir interface and the black dots correspond to the Fe atoms on the Pd side.

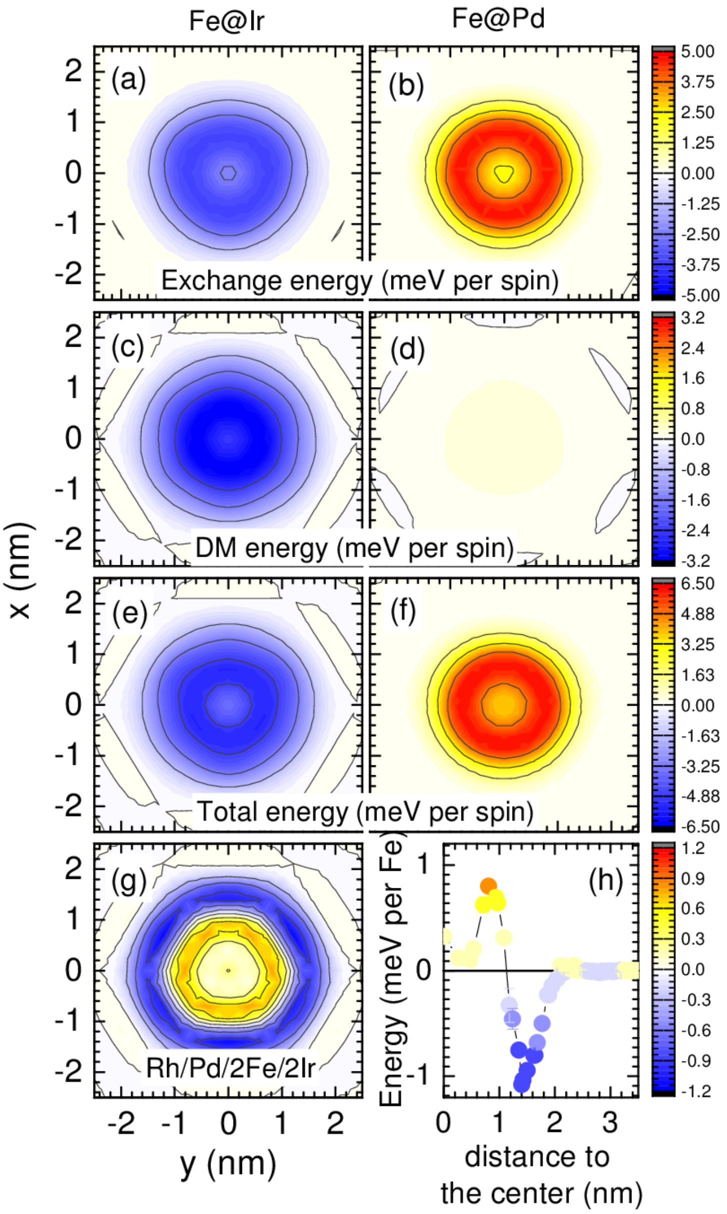

Supplementary Figure 6 | Site-resolved energy contributions to an isolated skyrmion in Rh/Pd/2Fe/2Ir at B = 3.5 T (cf. profile shown in Supplementary Figure 5) taking into account different interlayer exchange constants and DMI in the two Fe layers. (a,b) Site-resolved exchange energy in the Fe@Ir and in the Fe@Pd layer, respectively. (c,d) same as (a,b) for the DM energy. (e,f) same as (a,b) for the total energy. (g) site-resolved total energy of the Fe bilayer. (h) Total energy averaged over the δth neighbor shell. The reference of energy is set to the energy of the FM state.

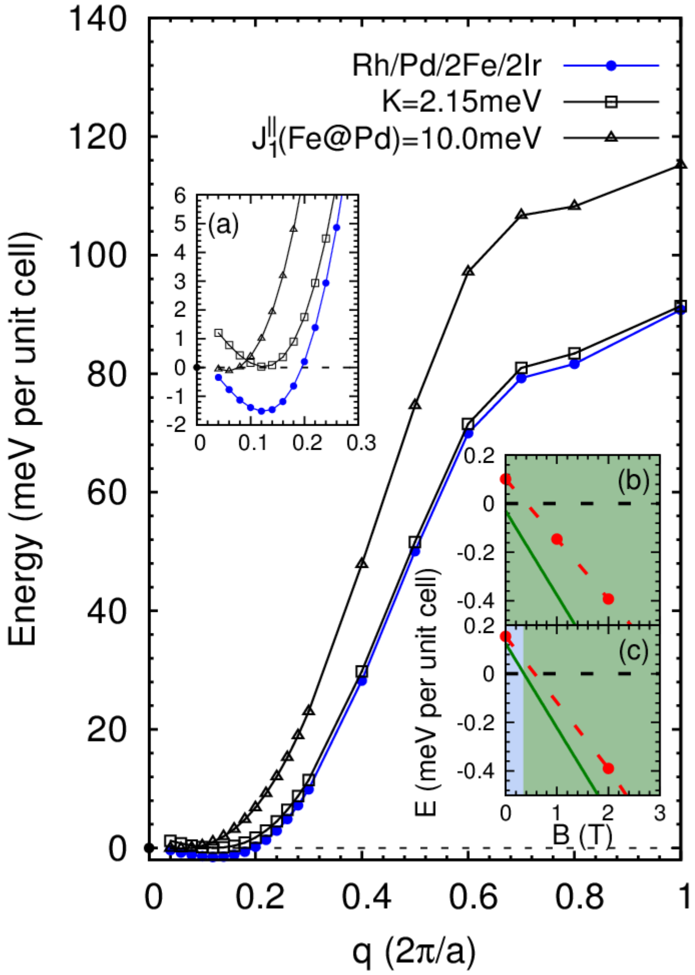

Supplementary Figure 7 | Energy dispersion of spin spirals in the $\bar{\Gamma}-\bar{K}$ direction of the Brillouin zone considering exchange interaction, DMI as well as the magnetocrystalline anisotropy. The blue curve is the dispersion for the perfect Rh/Pd/2Fe/2Ir multilayer system while for the black curve with triangles the nearest-neighbor exchange interaction within the Fe layer adjacent to Pd has been increased to $J_1^{\parallel,\mathrm{Fe@Pd}} = 10$ meV and for the black curve with squares the anisotropy has been increased to $K = 2.15$ meV. Inset (a) shows a magnified view of the main figure near $q = 0$. The phase diagrams for the scenarios with increased anisotropy, i.e. $K = 2.15$ meV, and high exchange ($J_1^{\parallel,\mathrm{Fe@Pd}} = 10$ meV) are given in the insets (b) and (c), respectively. The color coding follows Fig. 2 of the main paper.

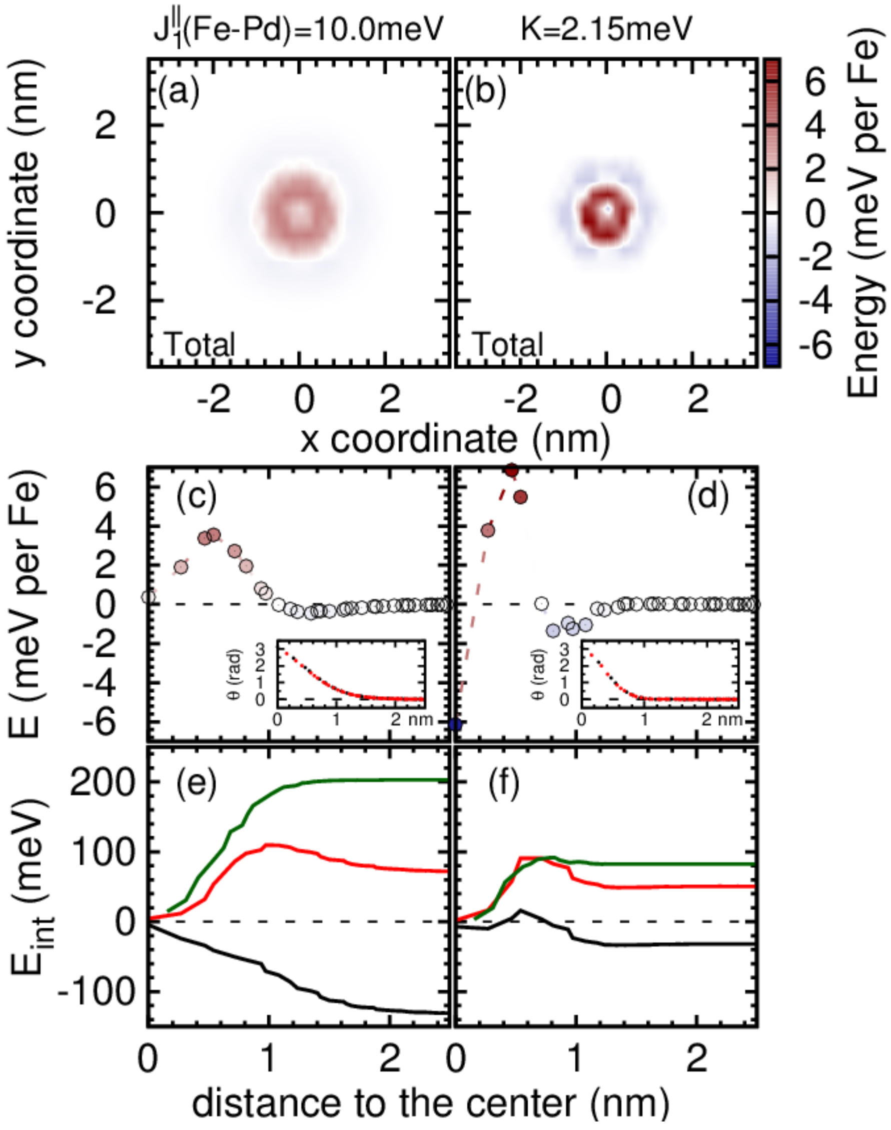

Supplementary Figure 8 | Total energy of an isolated skyrmion at $B = 0$ T in the case of Rh/Pd/2Fe/2Ir for $J_1^{\|,\mathrm{Fe@Pd}} = 10$ meV (left column) and $K = 2.15$ meV (right column). The center of the skyrmion is located at (0,0). The top panels show the site-resolved total energy. The middle rows show the total energy averaged over the δth neighbor shell; insets show the skyrmion profile. The bottom panels show the total energy summed over the shells from the skyrmion center up to the radius r for the Fe bilayer (red), Fe@Pd (green) and Fe@Ir (black).

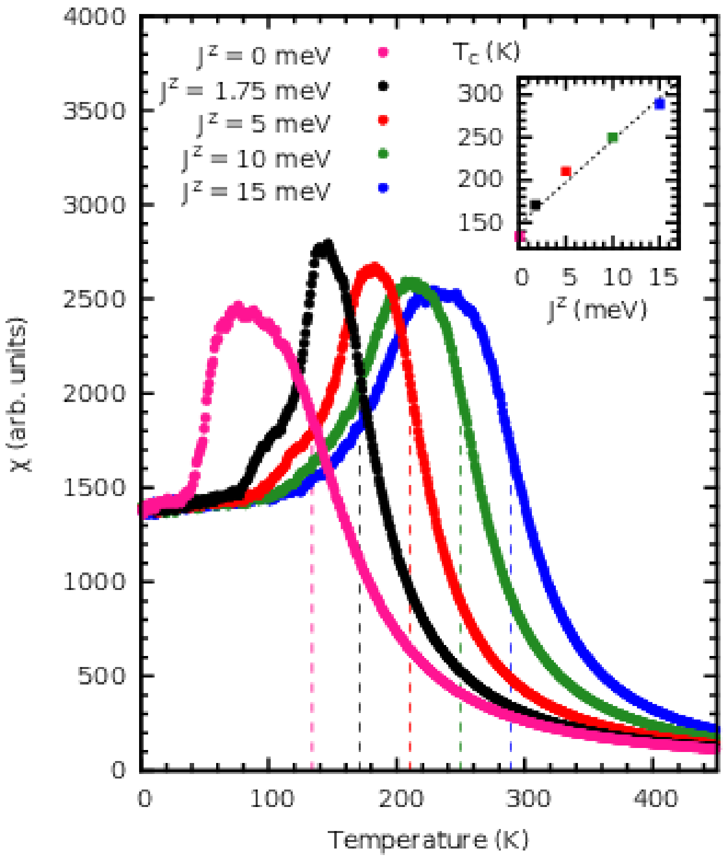

Supplementary Figure 9 | Temperature dependence of the susceptibility χ of the multilayer system [Rh/Pd/2Fe/2Ir] as a function of the strength of the exchange coupling between adjacent Fe bilayers. Dashed lines indicate the inflection point of the curves which has been used to determine the transition temperatures. The inset shows the transition temperature T_c as a function of the inter-bilayer exchange coupling J^z. The dotted line in the inset is a guide for the eye.

Supplementary Table 1 | Interlayer distances in the ultra-thin film and multilayer systems

	$d_{\text{Rh-Rh}}$	$d_{\text{Rh-Fe}}$	$d_{\text{Fe-Fe}}$	$d_{\text{Fe-Ir}}$	$d_{\text{Ir-Ir}}$	$d_{\text{Ir-Rh}}$
2Rh/2Fe/2Ir	2.27	2.09	1.99	2.11	2.29	2.11
	$d_{\text{Pd-Pd}}$	$d_{\text{Pd-Fe}}$	$d_{\text{Fe-Ir}}$	$d_{\text{Ir-Ir}}$		
2Pd/Fe/Ir(111)	2.24	2.01	2.06	2.19		

Interlayer distances obtained after structural relaxation of the Rh/Pd/2Fe/2Ir multilayer system and the ultra-thin film 2Pd/Fe/Ir(111) from Ref. [4]. All distances are given in Å.

Supplementary Table 2 | Inter- and intralayer exchange coupling constants

$J_1^{\parallel}$	$J_2^{\parallel}$	$J_3^{\parallel}$	$J_4^{\parallel}$	$J_1^{\perp}$	$J_2^{\perp}$	$J_3^{\perp}$	$J_4^{\perp}$
3.86	0.53	−3.04	0.11	24.73	0.18	−1.49	1.06

Exchange constants obtained from fitting the energy dispersion of spin spirals calculated via DFT for Rh/Pd/2Fe/2Ir (see Supplementary Note 2 for details). The coefficients are given in meV.

Supplementary Table 3 | Layer resolved intralayer exchange coupling constants

α	$J_1^{\parallel,\alpha}$	$J_2^{\parallel,\alpha}$	$J_3^{\parallel,\alpha}$	$J_4^{\parallel,\alpha}$
Fe@Ir	−1.70	0.36	−1.53	0.03
Fe@Pd	3.67	0.56	−0.84	0.26
	$J_1^{\perp}$	$J_2^{\perp}$	$J_3^{\perp}$	$J_4^{\perp}$
Fe-Fe	27.42	0.24	−0.40	−0.60

Exchange constants obtained from fitting the energy dispersion of spin spirals calculated via DFT for Rh/Pd/2Fe/2Ir (see Supplementary Note 4 for details). In the first two lines the intralayer exchange couplings within the Fe layer adjacent to the Ir layer and within the Fe layer adjacent to the Pd layer are given. In the last line the interlayer exchange coupling constants between the two Fe layers are given. The coefficients are given in meV.

Supplementary Note 1 | Computational details

We have performed density functional theory (DFT) calculations applying the full potential linearized augmented plane wave (FLAPW) method as implemented in the FLEUR code (www.flapw.de). For all transition-metal interfaces, we have used the theoretical Ir lattice constant of 3.82 Å. The structural relaxation was carried out using a mixed functional combining the local density approximation (LDA) and the generalized gradient approximation (GGA) introduced in Ref. 1 to treat systems of 3d- and 5d-transition metals. The mixed functional takes into account the gradient correction in the interstitial region and in the muffin tin (MT) spheres of the 3d atoms and neglects it in the MT of the 4d and 5d species. Therefore, the resulting functional treats the MT of the 3d atoms and the interstitial region in GGA[2], and the MT of the heavier atoms in LDA[3]. For the multilayer systems Pd/Fe/Pd/Fe/2Ir and 2Pd/Fe/2Ir we used the relaxed interlayer distances from our film calculations[4]. For the multilayer systems Rh/Rh_xPd_{1-x}/2Fe/2Ir, we applied the structural relaxations obtained for the system 2Rh/2Fe/2Ir. For the relaxations we used a cutoff parameter for the basis functions of $k_{\max} = 3.8\,\text{a.u.}^{-1}$ and $(16 \times 16 \times 4)$ k-points in the Brillouin zone (BZ). The structural parameters obtained after optimization are given for two representative systems in Supplementary Table 1.

The energy dispersions $E(\mathbf{q})$ of spin spirals for the multilayers were obtained using a cutoff parameter for the basis functions of $k_{\max} = 3.8\,\text{a.u.}^{-1}$ and $(32 \times 32 \times 4)$ k-points in the BZ. The contribution of the Dzyaloshinskii-Moriya interaction (DMI) was calculated using first order perturbation theory[5]. The magnetocrystalline anisotropy energy (MAE) has been obtained using the force theorem starting from the converged scalar-relativistic charge density. We used a cutoff parameter for the basis functions of $k_{\max} = 4.0\,\text{a.u.}^{-1}$ and $(32 \times 32 \times 4)$ k-points in the BZ.

Supplementary Note 2 | Energy dispersion of spin spirals

We consider flat spin spirals in which the magnetic moments are confined in a plane with a constant angle between moments at adjacent lattice sites propagating along high symmetry directions of the surface[6]. Such a spin spiral can be characterized by a wave vector $\mathbf{q}$ from the two-dimensional Brillouin zone (BZ) and the magnetic moment of an atom at site $\mathbf{R}_i$ is given by $\mathbf{M}_i = M\,(\sin(\mathbf{q}\cdot\mathbf{R}_i), \cos(\mathbf{q}\cdot\mathbf{R}_i), 0)$ and the size of the magnetic moment M. From the calculated energy dispersion $E(\mathbf{q})$ of spin spirals we can determine the exchange interactions J_{ij} as described below.

In Supplementary Figure 1 we display the energy dispersion $E(\mathbf{q})$ of spin spirals close to the ferromagnetic state obtained using DFT for the multilayer systems. For the ultra-thin film systems the energy dispersions can be found in Ref. 4. Spin-orbit coupling has not been taken into account for these calculations. The rise or drop of the dispersion curve in the vicinity of the ferromagnetic state, i.e. $q = 0$, is captured by the effective exchange constant J_{eff} shown in Fig. 1 of the main paper. The value of the spin spiral vector q at which the energy has been obtained for E_{SS} is also indicated by a dashed line.

We have fitted the exchange dispersion curve to the spin Hamiltonian

$$H = -\sum_{ij} J_{ij}^{\parallel} (\mathbf{m}_i \cdot \mathbf{m}_j) - \sum_{ij} J_{ij}^{\perp} (\mathbf{m}_i \cdot \mathbf{m}_j), \qquad (1)$$

where the first sum runs over sites within each Fe layer and $J_{ij}^{\parallel}$ are the intralayer exchange coupling constants and the second sum runs over sites in different layers with $J_{ij}^{\perp}$ parameterizing the exchange between the two Fe layers. The intralayer contributions, $J_{ij}^{\parallel}$, are obtained as in the monolayer case[7]. Due to the loss of inversion symmetry which results from the presence of the second iron layer, the derivation for the interlayer part, $J_{ij}^{\perp}$, differs slightly due to a phase shift to the atoms in the neighboring plane as given below. We can write the expression of the interlayer exchange energy contribution by inserting the magnetization for a flat spin spiral

$$H^{\perp} = -\sum_{\delta} J_{\delta}^{\perp} \sum_{\mu} \cos(\mathbf{q} \cdot \mathbf{R}_{\delta_\mu}), \qquad (2)$$

where $\mathbf{R}_{\delta_\mu}$ is the position of the atom μ in the δth nearest neighbor shell and $\mathbf{q}$ is the propagation vector of the spiral in units of $2\pi/a$. There are three magnetic first nearest neighbor atoms in the neighboring plane at the positions $\left\{\left(\frac{a}{2}, \frac{a}{2\sqrt{3}}\right), \left(-\frac{a}{2}, \frac{a}{2\sqrt{3}}\right), \left(0, -\frac{a}{\sqrt{3}}\right)\right\}$, which gives in Cartesian coordinates

$$H_1^{\perp} = -J_1^{\perp}\left(\cos\left(\frac{aq_y}{\sqrt{3}}\right) + 2\cos\left(\frac{aq_x}{2}\right)\cos\left(\frac{aq_y}{2\sqrt{3}}\right)\right) \qquad (3)$$

We can continue this procedure in order to calculate the first four $J_{\delta}^{\perp}$. This yields the expressions:

$$H_2^{\perp} = -J_2^{\perp}\left(\cos\left(\frac{2aq_y}{\sqrt{3}}\right) + \cos\left(aq_x + \frac{aq_y}{\sqrt{3}}\right) + \cos\left(\frac{1}{3}\left(aq_x - 3\sqrt{3}aq_y\right)\right)\right) \qquad (4)$$

$$H_3^{\perp} = -2J_3^{\perp}\left(\cos\left(\frac{3aq_x}{2}\right)\cos\left(\frac{aq_y}{2\sqrt{3}}\right) + \cos(aq_x)\cos\left(\frac{2aq_y}{\sqrt{3}}\right) + \cos\left(\frac{aq_x}{2}\right)\cos\left(\frac{5aq_y}{2\sqrt{3}}\right)\right) \qquad (5)$$

$$H_4^{\perp} = -2J_4^{\perp}\left(\cos(2aq_x)\cos\left(\frac{aq_y}{\sqrt{3}}\right) + \cos\left(\frac{3aq_x}{2}\right)\cos\left(\frac{5aq_y}{2\sqrt{3}}\right) + \cos\left(\frac{aq_x}{2}\right)\cos\left(\frac{7aq_y}{2\sqrt{3}}\right)\right) \qquad (6)$$

The best fit of the energy dispersion obtained with four $J^{\parallel}$ and four $J^{\perp}$ is given in Supplementary Table 2. Note, that we have assumed the intralayer exchange interactions to be the same within each Fe layer. We have tested the effect of this assumption by allowing for different values of $J_1^{\parallel,\alpha}$ in both

layers α and found that a variation of $J_1^{\|,\alpha}$ in the range of ± 1 meV (keeping $J_1^{\|} = \sum_\alpha J_1^{\|,\alpha}$ constant) neither changes the obtained phase diagram nor the skyrmion profile qualitatively. For a further discussion on separate intralayer exchange couplings within the two Fe layers see Supplementary Note 4.

Supplementary Note 3 | Exchange and DMI in Rh/Pd/2Fe/2Ir

We have calculated the energy dispersion $E(\mathbf{q})$ of spin spirals for Rh/Pd/2Fe/2Ir along the high-symmetry lines $\bar{\Gamma} - \bar{K}$ and in the $\bar{\Gamma} - \bar{M}$ directions of the two-dimensional BZ. As seen in the top panel of Supplementary Figure 2 the spin spiral dispersion curve is very flat close to the $\bar{\Gamma}$-point ($q = 0$, ferromagnetic state) without showing a spin spiral minimum driven by exchange interaction (blue curve). Upon including spin-orbit coupling (SOC) a right handed spin spiral is stabilized with a period of $\lambda \approx 2.25$ nm (red curve). We can make a layer-wise decomposition of the SOC contributions[5,8] as shown in the bottom panel of Supplementary Figure 2.

The total DMI of 1.3 meV (red line) mainly arises from the contribution of 1.5 meV from the Ir layers (black dots) while the Rh/Pd layers have a smaller contribution to the DMI of opposite sign amounting to -0.3 meV. The contribution of the double layer Fe has a much smaller magnitude of 0.1 meV. In our spin Hamiltonian, we consider the DMI to arise only at the Fe atoms adjacent to the Ir layers while we set DMI zero for the Fe layer at the Rh/Pd interface.

To check our approximation, we have performed Monte-Carlo calculations with competing DMI at the two interfaces. We have chosen values of 1.5 meV for the Fe atoms at the Ir interface and an opposite DMI with a value of -0.3 meV for the Fe atoms at the Pd interface. At a field of 3.5 T, which is also chosen for the calculations presented in Fig. 3 of the main paper, we find a skyrmion which is embedded in the two Fe layers with a profile as in Fig. 3. The only difference is a slight distortion from the circular shape. Therefore, our approximation of treating DMI at only one interface is well fulfilled for our systems.

Note, that the four-spin interaction which couples spin spirals into the nanoskyrmion lattice of Fe/Ir(111)[9] is short ranged. Therefore, it is most effective if the energy minimum of spin spirals occurs for short periods, e.g. $\lambda = 1$ nm in the case of Fe/Ir(111). For the multilayer systems discussed in this paper the period or in an external field the skyrmion diameter is controlled by the 4*d*/Fe interface, e.g. we obtain $\lambda = 2.25$ nm for Rh/Pd/2Fe/2Ir. Thereby, we go into a regime in which the four-spin interaction, which acts on the atomic scale and becomes strong for fast rotating spirals, plays a minor role and cannot enforce a zero-field atomic-scale skyrmion lattice.

Supplementary Note 4 | Variation of intralayer exchange couplings in the bilayer

In the case of a multilayer, the local environment of each of the magnetic layers may result in different intralyer exchange interactions. For example, in the Fe bilayers which we consider one of the layers is adjacent to a 4*d*- and the other one to a 5*d*-transition metal layer. In order to check the influence of the different hybridization at these layers on the exchange interaction, we have performed spin spiral calculations based on DFT in which a spiral propagates only in one of the Fe layers while the spins in the other one are kept in a ferromagnetic configuration and perpendicular to the plane of the spin spiral. This implementation within the FLEUR code allows the calculation of the energy dispersion curve in each of the magnetic layers separately and to extract the intralayer exchange couplings $J^{\parallel}$ within each layer for a vanishing interlayer coupling $J^{\perp} = 0$.

Supplementary Figure 3 shows the results of such DFT calculations for the multilayer Rh/Pd/2Fe/2Ir. The energy dispersion of a homogeneous spin spiral in both Fe layers (red) has been added for comparison (cf. Supplementary Figure 1). When the spins are fixed in the Fe@Pd layer and a homogeneous spin spiral propagates in the Fe@Ir layer (blue), the energy becomes maximum at the $\overline{\Gamma}$-point and minimal at the $\overline{K}$-point indicating an AFM ground state. The exchange interaction will favor a fast rotating spin spiral in this layer. On the other hand, when a homogeneous spin spiral propagates in the Fe@Pd layer, the $\overline{\Gamma}$-point is the energy minimum. The magnetic exchange favors a FM ground state in this layer. When a homogeneous spin spiral propagates in both Fe layers simultaneously, the interaction between the Fe layers, $J^{\perp}$, becomes significant. Although the intralayer exchange $J^{\parallel}$ favors a different ground state in each of the Fe layers taken separately, the energy dispersion curve of a homogeneous spin spiral in both Fe layers simultaneously has a FM ground state. Therefore, the interlayer exchange coupling plays a crucial role in such multilayer structures. Close to the $\overline{\Gamma}$-point, the energy rises very slowly that confirms the presence of exchange frustration in the Fe layers. If we take DMI into account a spin spiral ground state occurs.

In order to quantify the intra- and interlayer exchange interactions, we have fitted the first-principles calculations shown in Supplementary Figure 3 to an extended Heisenberg model. The fitting coefficients are given in Supplementary Table 3. We have obtained layer-resolved interlayer coefficients $J^{\parallel,\alpha}$ by fitting the energy dispersion curve of a homogeneous spin spiral propagating independently in the layers $\alpha =$Fe@Ir and $\alpha =$Fe@Pd. At the Ir interface $J_1^{\parallel,\mathrm{Fe@Ir}} = -1.70$ meV reveals an AFM ground state. In this layer, the frustration of the exchange energy is important since the $J^{\parallel,\mathrm{Fe@Ir}}$ do not decay exponentially and $J_3^{\parallel,\mathrm{Fe@Ir}} = -1.53$ meV is very similar to the value of $J_1^{\parallel,\mathrm{Fe@Ir}}$. When a homogeneous spin spiral propagates in the Fe@Pd layer, the situation is more conventional. The neareast-neighbor intralayer exchange constant $J_1^{\parallel,\mathrm{Fe@Pd}} = 3.67$ meV, i.e. positive,

which is in accordance with a FM ground state. In order to obtain $J^{\perp}$, we have fitted the energy dispersion curve for a homogeneous spin spiral propagating in both Fe layers simultaneously while fixing the values of the intralayer exchange couplings $J^{\parallel,\alpha}$ extracted before. When the magnetic interaction between the Fe layers is taken into account, we find a FM ground state for Rh/Pd/2Fe/2Ir and an interlayer coupling of $J_1^{\perp} = 27.42$ meV. This value is in very good agreement with the value of $J_1^{\perp} = 24.73$ meV given in Supplementary Table 2.

We have performed Monte-Carlo simulations using the intra- and interlayer exchange constants from Supplementary Table 3 in order to explore the stability of the spin spiral, the skyrmion lattice and the FM phase as a function of the applied magnetic field. In these simulations, we have also taken different constants for the DMI at the Fe@Ir and Fe@Pd into account with values of 1.5 meV and -0.3 meV, respectively, as discussed in Supplementary Note 3. Qualitatively, the phase diagram is as the one presented in Fig. 2 of the main paper which was obtained based on assuming the same interlayer exchange constants in both Fe layers (cf. Supplementary Table 2) and DMI only at the Fe@Ir layer. The ground state is a spin spiral with a period of $\lambda \approx 2.3$ nm. The spin spiral phase is stable up to $B \approx 2$ T. Then a lattice of skyrmions with diameters of about 2.7 nm is stable up to $B \approx 4.1$ T as shown in Supplementary Figure 4.

The profile of the isolated skyrmion at a field of $B = 3.5$ T (Supplementary Figure 5) shows that the skyrmion diameter increases by around 1 nm as compared with the set of coefficients described in Supplementary Note 2. In order to analyze the stabilization mechanism of isolated skyrmions in Rh/Pd/2Fe/2Ir, we show in Supplementary Figure 6 the site-resolved exchange, DM, total energy and the radially averaged total energy of an isolated skyrmion at $B = 3.5$ T (corresponding to Figs. 3 and 4 of the main paper and Supplementary Figure 5). In the Fe@Ir layer (Supplementary Figure 6a), the exchange favors a fast rotating spin spiral. Therefore, the exchange energy of a non-collinear spin structure always gains energy as compared with the FM background. This is opposite to the situation in the Fe@Pd layer (Supplementary Figure 6b) where a collinear arrangement of the spins is preferred as expected from the interlayer exchange constants (cf. Supplementary Table 3).

The DM energy in Fe@Ir favors a right-handed non-collinear spin structure and therefore favors the skyrmions shown in Fig. 3 of the main paper. The amplitude of the DMI is 1.5 meV in this layer and therefore, the DM contribution shown Supplementary Figure 6c has a strong stabilizing effect. On the other hand, the DM contribution of Fe@Pd is very small (-0.3 meV) and favors a left-handed skyrmion, which is the skyrmion of the other rotational sense. This contribution is negligeable and almost shows no contrast in Supplementary Figure 6d.

When all the energy contributions are taken into account (exchange, DMI, anisotropy and Zeeman energy), the Fe@Ir contribution is the stabilizing layer of the right-handed skyrmion (Supplementary Figure 6e). On the other hand, the Fe@Pd layer has a strong destabilizing contribution of the same amplitude as compared with the contribution of the Fe@Ir layer (Supplementary Figure 6f). The strong interlayer exchange coupling $J_1^{\perp}$ which favors collinear spin arrangement between the layers is, surprisingly, always a stabilizing term due to the profile of the right-handed skyrmion of Supplementary Figure 5 that shows a strong embedment of the skyrmion within the two Fe layers. The difference of energy contribution between each of the layers can only be attributed to their different energy dispersion curves shown in Supplementary Figure 3.

When the two contributions of the layer resolved total energies are added, the energy density distribution of a right-handed skyrmion displays an oscillatory behavior as shown in Supplementary Figure 6g. The total energy averaged over the δth neighbor shell is shown in Supplementary Figure 6h. The core of the skyrmion, which corresponds to the central atom ($r = 0$ nm) up to $r \approx 1$ nm, is now a destabilizing region due to the Fe@Pd layer energy contribution. The skyrmion is stable due to its stabilizing tail region that starts at $r \approx 1$ nm. In that region, both the exchange energies of the Fe@Ir and the Fe@Pd layers are vanishing. The stabilizing mechanism is the DM interaction. From the integration of the energy density of Supplementary Figure 6h, the energy gain of an isolated skyrmion can be calculated. At $B = 3.5$ T, an isolated skyrmion gains 51.0 meV as compared with the FM background although each of the magnetic layers do not have the same ground state. This suggests that isolated skyrmion systems can be hosted in a broad range of magnetic multilayers where the magnetic ground state in each of the magnetic layers considered by themselves must not be identical.

Supplementary Note 5 | Stability with respect to variations of J_{eff} and K

Intermixing at the 4*d*/Fe interface can affect the exchange interactions in the Fe layers. We have performed DFT calculations for the Rh/$Pd_{0.66}Fe_{0.33}$/$Pd_{0.33}Fe_{0.66}$/Fe/2Ir multilayer system as an ordered alloy in a $\sqrt{3} \times \sqrt{3}$ unit cell. By performing spin spiral calculations we have obtained the effective exchange constant which is $J_{\mathrm{eff}} \approx 9$ meV. Compared to the multilayer Rh/Pd/2Fe/2Ir with a perfect interface with $J_{\mathrm{eff}} = 1$ meV the system apparently becomes much more ferromagnetic. In the spirit of Fig. 1 of the main paper, one can decrease the effective exchange for the alloyed multilayer by increasing the amount of Rh in the interface layers.

In order to account for such an increase of the exchange interaction of an alloyed multilayer in our spin dynamics simulation, we have increased the parameter $J_1^{\parallel}$ for the Fe layer at the Pd interface in our spin dynamics simulation to a value of 10 meV. We can relate the change of $J_1^{\parallel}$ in our simulations to the effective exchange constant by performing a fit to the spin spiral energy dispersion shown in

Supplementary Figure 7 around $q = 0$ which leads to a value of $J_{\text{eff}} = 4$ meV. As seen in Supplementary Figure 7, this leads to a faster increase of the energy close to the ferromagnetic state, i.e. $q = 0$, and the energy minimum disappears. Thus the ferromagnetic state becomes the ground state. Therefore, this value of J_{eff} represents the limiting case in which a skyrmion lattice does not appear in the phase diagram as shown below. We conclude that for all systems in Fig. 1 of the main paper with $J_{\text{eff}} < 4$ meV stable skyrmion formation is possible. However, for very large negative values of J_{eff} gigantic magnetic fields may be necessary.

Another influence of the alloying can be a change of the anisotropy K. This can be studied in the case of Rh/Rh$_x$Pd$_{1-x}$/2Fe/2Ir multilayers, where the uniaxial anisotropy increases monotonously from 0.7 meV for $x = 0$ to 1.5 meV for $x = 1$. We take this effect into account in a second scenario for the limiting case, i.e. when the energy minimum disappears due to a large K of 2.15 meV. The other exchange constants as well as the DMI are left unchanged from the perfect Rh/Pd/2Fe/2Ir multilayer system.

Phase diagram. In order to illustrate the effect of the variation of K and $J_1^{\parallel}$ (or J_{eff}, cf. previous section) on the energy of the multilayer depending on the spin-spiral vector, $\mathbf{q}$, we show in Supplementary Figure 7 the dispersion curve corresponding to the three cases. The blue curve is the reference case where all the coefficients were obtained from our first principles calculations for the perfect Rh/Pd/2Fe/2Ir multilayer. As explained in the main body of this paper, in this case the DMI stabilizes the spin spiral ground state. The minimum of the dispersion curve is deep enough to overwhelm the contribution of the anisotropy. When only the anisotropy is increased, the blue curve changes toward the black curve with square symbols, indicating the case for which $K = 2.15$ meV. In that case, the anisotropy has been tripled and compensates exactly the DM energy. The dispersion curve shows two degenerate minima: One at the $\bar{\Gamma}$-point, which corresponds to the FM state, and one at $q = 0.1 \times 2\pi/a$ in the $\bar{\Gamma} - \bar{K}$ direction.

In the other scenario, we kept $K = 0.6$ meV but we increased $J_1^{\parallel,\text{Fe@Pd}}$ to 10 meV which corresponds to an effective exchange interaction of $J_{\text{eff}} = 4$ meV (triangles in Supplementary Figure 7). As a consequence, the period of the spin spiral increases dramatically as the exchange energy increases and compensates the DMI. Then there is an evolution from the blue curve towards the black curve with triangles. As compared with the case of strong anisotropy, we do not go through two degenerate minima when the exchange is increased.

Analysis of the energy contributions. Even in the limiting cases discussed above in which there is no stable skyrmion lattice in the phase diagram, metastable skyrmions can form. We have analyzed the

energetical stability of such metastable isolated skyrmions in both cases, i.e. of increased exchange stiffness and of increased magnetic anisotropy. We focus on the situation of vanishing external magnetic field where the energy difference with respect to the ferromagnetic state is smallest. Supplementary Figure 8 shows the site-resolved total energy of the isolated skyrmion per Fe atom in the case of $J_1^{\parallel,\mathrm{Fe@Pd}} = 10$ meV (left column) and $K = 2.15$ meV (right column). In both cases the site-resolved energy has a similar behavior with a minimum at the center of the skyrmion, then a maximum due to exchange, and finally a small negative contribution due to the DMI. Although the stabilizing forces have the same behavior as in the case shown in Fig. 4 of the main text, the total energy averaged over the δth neighbor shell, shown the middle row of panels of Supplementary Figure 8, shows a strongly frustrated behavior in the case of strong anisotropy: While the anisotropy strongly disfavors the magnetic structure near the center, due to the DMI some energy is gained at the edge of the skyrmion.

When $J_1^{\parallel,\mathrm{Fe@Pd}} = 10$ meV (corresponding to an J_{eff} of 4 meV) the spin spiral minimum of the exchange dispersion curve is compensated by the strong FM behavior of the Fe@Pd interface, which cannot be compensated by the DMI. The situation is different in the case of strong anisotropy, where a minimum of the dispersion curve is created by DMI and exchange, but it is compensated by the anisotropy. This creates, as shown Supplementary Figure 8(d), a strong frustration of the spin structure. The site-resolved energy exhibits a deep minimum at $r = 0$ nm, due to the addition of a strong out-of-plane anisotropy and DMI, and a high maximum at $r = 0.5$ nm. The insets of Supplementary Figure 8(c) and (d) show the θ profile of the isolated skyrmion. When the exchange is increased the isolated skyrmion is barely changing size as compared to the case shown in Fig. 3 of the main text. On the other hand, an increase of the anisotropy reduces drastically the size of the skyrmion (from approx. 3 nm to 2 nm) in agreement with previous studies[10] that implies an increase of the energy per spin. Although the total energy summed over all spins within the skyrmion is 51 meV in the case of large anisotropy and 71 meV in the case of large exchange (cf. Supplementary Figure 8(e,f)), the energy per spin is 14 μeV and 11 μeV, respectively. While the total energy of the skyrmion at $B = 0$ T is lower in the case of high anisotropy, the frustration in the spin lattice is higher.

Supplementary Note 6 | Inter-bilayer exchange coupling and transition temperature

Using DFT and Monte-Carlo (MC) simulations we have also studied the effect of the exchange coupling between adjacent Fe bilayers in our multilayers, i.e. the inter-bilayer exchange coupling. In order to take the exchange coupling between adjacent Fe bilayers into account we have extended the Hamiltonian of Supplementary Equation (1) by the term

$$H_{\text{inter}} = -J^z \sum_i \sum_{q=-1,+1} (\mathbf{m}_i^p \cdot \mathbf{m}_i^{p+q})$$

for every Fe bilayer. Here $\mathbf{m}_i^p$ denotes the spin at site i within the Fe bilayer p. From DFT calculations we obtain $J^z = 1.75$ meV for multilayers from a repetition of the sandwich [Rh/Pd/2Fe/2Ir] and $J^z = 3.75$ meV for multilayers based on [2Pd/2Fe/2Ir].

We have determined the Curie temperature, T_c, at $B = 0$ T via parallel tempering MC simulations. The starting spin configuration is a superlattice of (20×20) spins per Fe layer which contains two spin spiral periods along the $\bar{\Gamma} - \bar{M}$ high symmetry line. The multilayer system consists of 6 Fe bilayers. We used periodic boundary conditions in the perpendicular and in the lateral directions. We have checked for the bilayer that increasing the cell size e.g. to (100×100) spins or increasing the number of bilayers in our multilayer simulation shows no difference except for enhanced noise for smaller cell sizes. The curves were obtained with a parallel MC simulation which computes simultaneously 456 spin configurations at different temperatures between 1 and 451 K. We have used $5 \cdot 10^4$ swaps between spin configurations of adjacent temperatures (average steps). Each spin configuration is thermalized for $2.4 \cdot 10^5$ Metropolis MC steps after each swap. We have used the coefficients of Rh/Pd/2Fe/2Ir for the magnetic exchange coupling within one Fe bilayer and we have varied the inter-bilayer exchange coupling J^z from 0 meV to 15 meV. Our estimation of T_c does not take into account dipole-dipole interaction.

We have defined the Curie temperature from the inflection point of the magnetic susceptibility χ, i.e. from $\frac{\partial^2 \chi}{\partial T^2} = 0$. The susceptibility χ is obtained from

$$\chi = \frac{\langle m^2 \rangle - \langle m \rangle^2}{k_B T}$$

The T_c extracted from the peak of the specific heat or the total energy inflection point is in relatively good agreement (± 10 K) with the method described above.

Supplementary Figure 9 shows the susceptibility as a function of temperature for various values of the inter-bilayer exchange coupling J^z. Clearly the peak of χ shifts to higher temperatures with increasing J^z which indicates an increase of T_c. The inset of Supplementary Figure 9 shows the scaling of T_c with the inter-bilayer exchange coupling. The case of $J^z = 0$ meV (pink) corresponds to the T_c of an isolated Fe-bilayer of the system [Rh/Pd/2Fe/2Ir]. For the multilayer built from [Rh/Pd/2Fe/2Ir], the transition temperature rises to $T_\mathrm{c} \approx 170$ K. For the inter-bilayer exchange coupling strength of the

[2Pd/2Fe/2Ir] multilayer a T_c of approximately 200 K is obtained. By increasing the inter-bilayer exchange coupling in our MC simulations we find that transition temperatures close to room temperature become feasible. Such an increase of the exchange coupling in multilayers may be achieved, e.g. by varying the number or species of 4*d* or 5*d* layers within the unit cell.

The interlayer exchange coupling also stabilizes the alignment of skyrmions in adjacent Fe bilayers across the 4*d*/5*d* layers. Based on the value of J^z which we determined from DFT for [Rh/Pd/2Fe/2Ir] we find an energy loss of about 78 meV per skyrmion if the two skyrmions in the adjacent Fe bilayers are replaced by the ferromagnetic state.

Supplementary References

1. De Santis, M. *et al*. Structure and magnetic properties of MnPt(110)-(1×2): A joint x-ray diffraction and theoretical study. *Phys. Rev. B* **75**, 205432 (2007).
2. Perdew, J. P. *et al*. Atoms, molecules, solids, and surfaces: Applications of the generalized gradient approximation for exchange and correlation. *Phys. Rev. B* **46**, 6671-6687 (1992).
3. Vosko, S. H., Wilk, L. & Nusair, M. Accurate spin-dependent electron liquid correlation energies for local spin density calculations: a critical analysis. *Canadian Journal of Physics* **58**, 1200-1211 (1980).
4. Dupé, B., Hoffmann, M., Paillard, C. & Heinze, S. Tailoring magnetic skyrmions in ultra-thin transition-metal films. *Nature Communications* **5**, 4030 (2014).
5. Heide, M., Bihlmayer, G. & Blügel, S. Describing Dzyaloshinskii-Moriya spirals from first-principles. *Physica B: Condensed Matter* **404**, 2678-2683 (2009).
6. Kurz, Ph., Förster, F., Nordström, L., Bihlmayer, G. & Blügel, S. Ab initio treatment of noncollinear magnets with the full-potential linearized augmented plane wave method. *Phys. Rev. B* **69**, 024415 (2004).
7. Al-Zubi, A., Bihlmayer, G. & Blügel, S. Modelling magnetism of hexagonal Fe monolayers on 4*d* substrates. *Phys. Status Solidi B* **248**, 2242-2247 (2011).
8. Zimmermann, B., Heide, M., Bihlmayer, G. & Blügel, S. First-principles analysis of a homochiral cycloidal magnetic structure in a monolayer Cr on W(110). *Phys. Rev. B* **90**, 115427 (2014).
9. Heinze, S. *et al*. Spontaneous atomic-scale magnetic skyrmion lattice in two dimensions. Nature Phys. **7**, 713-718 (2011).
10. Bogdanov, A. & Hubert, A. Thermodynamically stable magnetic vortex states in magnetic crystals. *J. Mag. Mag. Mat.* **138**, 255-269 (1994).
